# A Novel Case of Tethered Cord in a Five-Month-Old Male With Pallister-Killian Syndrome

**DOI:** 10.7759/cureus.11240

**Published:** 2020-10-29

**Authors:** Michael J Gigliotti, Yaw Tachie-Baffour, Ryan J Jafrani, Jessica Lane, Elias Rizk

**Affiliations:** 1 Neurosurgery, Penn State Health Milton S. Hershey Medical Center, Hershey, USA

**Keywords:** tethered cord, pallister-killian syndrome, neurosurgery, detethering, pediatric

## Abstract

A five-month-old male presented with an incidentally found low-lying conus medullaris on ultrasound and subsequent MRI demonstrating its position at L4. Pre-operative examination findings included mild, global hypotonia and a coccygeal dimple without bladder or bowel abnormalities or spasticity. The patient underwent spinal cord untethering with a section of filum terminale and was discharged without complication following his procedure. Follow-up at one year revealed continued baseline hypotonia without further neurosurgical needs. This is the first reported case of tethered cord syndrome described in a patient with Pallister-Killian syndrome managed successfully with neurosurgical intervention.

## Introduction

Pallister-Killian syndrome (PKS, Online Mendelian Inheritance in Man® [OMIM®] #601803) is a rare cytogenetic condition first described by Pallister et al. in 1977, with the first affected infant reported by Teschler-Nicola and Killian in 1981 [1, 2]. Caused by the presence of extra copies of the short arm of chromosome 12, PKS is characterized by craniofacial dysmorphism (prominent forehead with bitemporal alopecia, hypertelorism, wide mouth, and abnormal ears), variable developmental delay and intellectual impairment, hypotonia, epilepsy, pigmentary skin anomalies, diaphragmatic hernia, congenital cardiac defects, and other systemic abnormalities [3, 4]. PKS has been previously reported to occur sporadically, with an estimated prevalence of 1/20,000 based on analysis of liveborn infants resulting from pregnancies with prenatally identified marker chromosome (mosaic isochromosome 12p) [5]. We present a novel case of a five-month-old male with Pallister-Killian syndrome and a low-lying conus medullaris with a tethered spinal cord subsequently managed by spinal cord untethering with sectioning of the filum terminale.

## Case presentation

**Figure 1:**
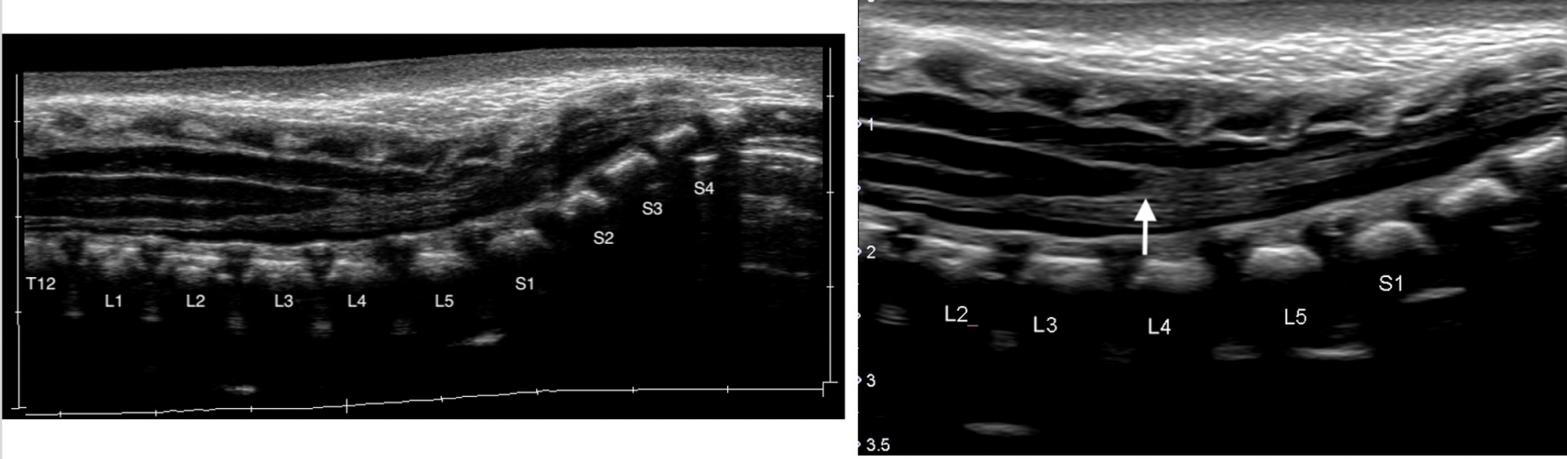
Ultrasound of the lumbar spine showing low-lying conus medullaris at the L4 vertebrae (white arrow)

A six-week-old male with a history of tetrasomy 12p consistent with Pallister-Killian syndrome born at 35-weeks' gestation to a 39-year-old G2P1 mother presented as a referral to the neurosurgical clinic with ultrasound findings of a low-lying conus at the end of the L4 lumbar vertebrae (Figure 1). The patient's ultrasound initially was obtained by his pediatrician due to a coccygeal dimple. 

**Figure 2:**
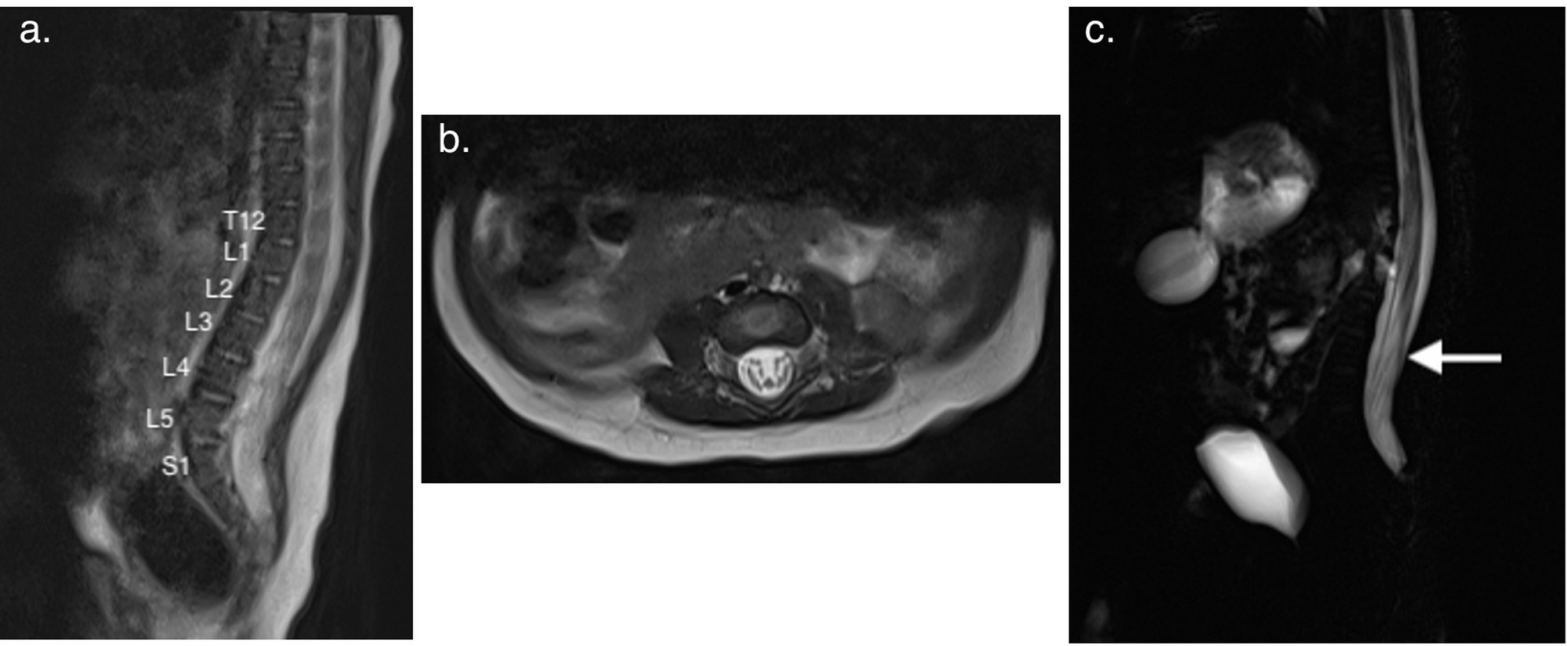
MRI T2-weighted imaging of the lumbar spine a. Sagittal imaging showing low-lying conus medullaris at L4. b. Axial imaging showing conus terminating at L4. c. Lumbar myelogram showing a low-lying conus with what appears to be filum terminale tethering (white arrow).

The patient also presented with magnetic resonance imaging (MRI) of the brain and cervical spinal cord due to poor trunk and head control, which demonstrated a small amount of hemorrhage in the caudal thalamic groove and periventricular calcifications. The patient's parents did not report any bowel or bladder problems prior to presentation to our clinic. On examination, the patient was awake and alert, with well-approximated cranial sutures and an anterior fontanelle that was soft and sunken. He was noted to have hypertelorism, high forehead, and sparse hair on his temples consistent with Pallister-Killian syndrome. The patient was able to move his upper and lower extremities; however, he did demonstrate mild, global hypotonia without associated spasticity. The patient was also found to have a coccygeal dimple buried within the gluteal cleft and overlying tip of the coccyx with overlying vellus hair. The patient did not have cutaneous markers of spinal dysraphism on his lumbosacral spine. The patient underwent a lumbar spine MRI to confirm the level and surrounding anatomy of the conus prior to determining whether definitive management of the tethered cord was indicated. This revealed a low-lying conus medullaris terminating behind L4 without skeletal abnormality of the lumbar spine or sacrum (Figure 2). 

The patient underwent surgical spinal cord untethering with section of the filum terminale at five months of age. Intraoperatively, a L5 lumbar laminectomy and midline durotomy were performed. The filum terminale was readily identifiable and appeared thickened. There was no evidence of filum lipoma or intradural tract concerning for spinal dysraphism intraoperatively. Primarily watertight closure of the dura was difficult due to it being thin and fragile. A DuraGen® onlay (Integra LifeSciences Corporation, Princeton, USA) and DuraSeal Exact Spine Sealant System® (Integra LifeSciences Corporation, Princeton, USA) were used to augment dural closure and to prevent cerebrospinal fluid (CSF) leakage. 

Post-operatively, the patient was kept flat overnight, his diet was advanced without difficulty, and he was noted to be voiding and stooling spontaneously. The patient was discharged on post-operative day one without complication. On one-year follow up, the patient was doing well from a neurosurgical standpoint. He was voiding on his own and his father described a normal urinary stream. The patient was undergoing physical therapy for right hip torsion and continued to have persistent hypotonia, however, he was able to move all four extremities spontaneously with effort against antigravity. 

## Discussion

Pallister-Killian syndrome (PKS) is characterized cytogenetically by mosaic tetrasomy 12p and the presence of supernumerary isochromosome composed of the short arms of chromosome 12 (I[12p]) [6]. Moreover, the PKS phenotype has been described in patients with trisomy 12p, and even patients with hexasomy 12p have been reported to have similar features [5, 7-9]. Due to a higher percentage of chromosomally normal cells and a lower degree of mosaicism present in lymphocytes compared to fibroblasts, cytogenetic data presented by Wilkens et al. confirmed that chromosomal analysis by karyotype on fibroblasts is a more sensitive way to detect i(12p) in affected individuals [3]. The mosaic distribution in PKS leads to significant phenotypical variability; however, PKS has been characterized as causing craniofacial dysmorphism with neonatal frontotemporal alopecia, hypertelorism, and low-set ears as well as kyphoscoliosis, severe intellectual disability, epilepsy, hypotonia, and systemic abnormalities [3, 4, 7-10].

Neurological abnormalities in PKS have been well documented. Specifically, hypotonia, seizures, structural brain abnormalities, and hearing and vision abnormalities have been observed. Blyth et al., in a retrospective study of 22 patients with PKS, reported that 90.9% of patients were hypotonic in the neonatal period, with 57.1% of patients remaining hypotonic as they grew older [9]. Despite this, variable spasticity and hypertonia has been seen in older individuals [2, 9]. Seizures and structural brain abnormalities have been reported with variability in the literature, with rates of 48% to 80% and 60% to 77.4%, respectively, in patients with PKS [2, 5-6, 9]. There appears to be variability in seizure presentation time from infancy to adulthood, and there is no specific seizure type associated with PKS. However, the most frequent seizure types observed in PKS includes myoclonic (40.9% - 56%), generalized tonic-clonic (22.7% - 48%), clustered tonic spasms (30%), and absence seizures (18.2%) typically occurring in the first four years of life [5, 9]. Similarly, there does not appear to be a signature pattern of radiographical or structural brain abnormalities in patients presenting with PKS. However, the most commonly reported radiographic finding in the literature is decreased brain parenchymal volume associated with ventricular, sulcal, and cisternal space enlargement representing volume loss (and/or hypoplasia) [7]. Other commonly reported abnormalities include corpus callosum dysgenesis, macrocephaly, and polymicrogyria (PMG), in addition to craniofacial abnormalities [6-7].

Despite the presence of neurological manifestations in patients with PKS, there have not been documented cases of patients presenting with tethered cord syndrome (TCS) in the literature. Classically, the tethered cord has been associated with a low-lying conus medullaris and presents as a constellation of symptoms and signs of motor and sensory neuron dysfunction attributable to abnormally increased tension on the spinal cord [11]. In the present case, the patient described did not have concomitant spinal dysraphism, and his coccygeal dimple is not typically associated with spinal bifida occulta or intraspinal anomalies. 

The presentation of a coccygeal dimple with concomitant tethered cord is very unusual, as coccygeal dimples have been reported as an innocent finding in four percent of the population [12]. Conversely, 16% to 17% of infants have been found to have a thickened finding on imaging [13]. Regardless, Weprin and Oakes demonstrated that the relative risk of neurologic infection or deficit with coccygeal dimples is exceedingly rare, and imaging is unnecessary [14]. We can reasonably conclude that the patient presented here had an incidental finding of a tethered cord with a coccygeal dimple. Interestingly, that may mean that a tethered cord is a variable phenotype of PKS. This has not been proven, however, as mosaic tetrasomy 12p with concomitant deletion at 15q11.2 (including breakpoints 1 and 2; BP1 and BP2) has been associated with scoliosis and ovoid-shaped vertebral bodies in infancy but not to tethered cord or neural tube defects [9, 11, 15-16]. Alternatively, the finding of the tethered cord reported here may be an incidental finding in a PKS patient.

The goal of operative intervention as its related to untethering surgery is to remove tension from the spinal cord without causing further trauma for the purpose of stabilizing symptomatology and function. Previous studies have shown that improved pain and stabilization of neurological decline are best achieved with surgical intervention, with rates approaching 100% and 80 to 90%, respectively [11]. However, the patient’s mild, global hypotonia found on pre-operative examination was likely related to his underlying genetic condition rather than the typical symptomatology seen in patients with symptomatic TCS. The role of untethering in asymptomatic patients with a low-lying conus found incidentally such as the one presented here, is controversial and has been significantly debated in the literature [17-20]. Lew and Kothbauer note that the arguments for prophylactic untethering in asymptomatic patients include a percentage of patients that may go on to develop symptomatic TCS, at which point the reversibility of symptoms is not guaranteed and the low morbidity for filum sectioning. Interestingly, untethering has been linked to stabilization (or improvement) in scoliotic curve progression with long-term rates noted at 43% to 63% [11]. This is important to note as patients with PKS have been documented to have associated scoliotic curvature of the spine. However, these stabilizing or improving scoliosis rates have been primarily reported in patients with myelomeningocele and not patients with asymptomatic presentations of low-lying conus.

## Conclusions

Pallister-Killian syndrome is a rare genetic condition that is associated with tetrasomy i(12p). Despite the presence of neurological manifestations of this genetic condition, we report the first case of tethered cord syndrome and subsequent surgical management with cord untethering in a patient with Pallister-Killian syndrome. Further study should be directed to determine whether there are other cases of spinal abnormalities associated with this unique mosaicism, and long-term study should be directed at whether spinal cord untethering is beneficial in patients presenting with TCS and Pallister-Killian syndrome. 
